# Bisphenol a Interferes with Uterine Artery Features and Impairs Rat Feto-Placental Growth

**DOI:** 10.3390/ijms22136912

**Published:** 2021-06-27

**Authors:** Laura Barberio, Luana Paulesu, Laura Canesi, Elena Grasselli, Maurizio Mandalà

**Affiliations:** 1 Department of Biology, Ecology & Earth Sciences, University of Calabria, 87036 Rende, Italy; laura.barberio90@gmail.com; 2 Department of Life Sciences, University of Siena, 53100 Siena, Italy; luana.riccipaulesu@unisi.it; 3 Department of Earth, Environment and Life Sciences (DISTAV), University of Genova, 16132 Genova, Italy; laura.canesi@unige.it (L.C.); elena.grasselli@unige.it (E.G.); 4 Department of Obstetrics, Gynecology and Reproductive Sciences, University of Vermont, Burlington, VT 05405, USA

**Keywords:** pregnancy, uterine arteries, maternal BPA contamination, endothelial dysfunction, nitric oxide, estrogen receptor, peroxisome proliferator-activated receptor ɣ

## Abstract

Bisphenol A (BPA) is a widespread environmental contaminant, found in human fluids and tissues. Maternal BPA exposure is associated with alterations in pregnancy outcomes. Because maternal uterine circulation plays a crucial role in normal placenta and fetal growth, we hypothesized that BPA compromises the function of uterine arteries (UAs) and fetoplacental development. Female rats were orally administered with BPA (2.5, 25 and 250 µg/kg/day) or with its vehicle (ethanol) for 30 days before pregnancy and during the first 20 days of pregnancy. To compare the effect of BPA in the reproductive vs. systemic circulation, it was tested on UAs and mesenteric arteries (MAs). Arteries were isolated and examined by pressure myography. Moreover, fetuses and placentas were weighed to provide an index of reproductive performance. In UAs of BPA-treated rats, lumen diameter, acetylcholine-relaxation and expressions of endothelial nitric oxide synthase 3 (*NOS3*), estrogen receptor α (*ERα*) and peroxisome proliferator-activated receptor ɣ (*PPARɣ*) were reduced. Conversely, no changes were observed in MAs. BPA treatment also reduced placental weights, while fetal weights were increased. For the first time, our results indicate that UAs represent a specific target of BPA during pregnancy and provide insight into the molecular mechanisms that underlie its negative effects on pregnancy outcomes.

## 1. Introduction

Bisphenol A (BPA), 2,2-bis (4-hydroxyphenyl) propane or 4,4′—isopropylidenediphenol (CAS number: 80-05-7) [1], is a synthetic chemical component of polycarbonate, a plastic used in the packaging of common consumer goods [2]. BPA is also a component of the epoxy resin present in food containers, in thermal papers and in many other objects in daily use [2]. During heating, in acidic or alkaline medium, BPA can be easily released from the polymer product and migrate into food, beverages and the environment [3]. Thus, it can be introduced into the human body in different ways: ingestion, inhalation and absorption [4,5,6,7], and is present in blood and urine at levels of 1–10 ng/mL [8,9,10]. Concentrations up to 22.3 ng/mL were observed in the blood of pregnant women [11,12]. BPA is also present in amniotic fluid [13], placenta and umbilical cord [14]. Because BPA belongs to a class of chemical compounds that interferes with the endocrine system—endocrine disrupting chemicals (EDC)—its presence has implications for human health and for women’s reproductive health [15].

In human pregnancy, BPA acts on the placenta, impairing its growth and development in the maternal uterus. In vitro studies on human choriocarcinoma cells, BeWo, showed that BPA upregulates the secretion of hCG and cell apoptosis, two markers of differentiation of the syncytiotrophoblast, the epithelial layer of chorionic villi [16,17]. By using the HTR-8/SVneo, a representative cell type of the extravillous trophoblast, it was demonstrated that BPA reduced the cell migration and invasion, without affecting the cell proliferation [18]. BPA also induces changes in membrane glucose type 1 transporter (GLUT-1), the primary placental transporter of glucose, in HTR-8/SVneo cells as well as in the placenta of rats fed with BPA [19].

In addition, several in vitro studies on human stromal endometrial cells have demonstrated that BPA can lead an alteration on markers of decidualization impairing the process of placentation in the maternal uterus [20,21,22]. These studies suggest that maternal contamination with BPA, during times of fertility and pregnancy, can affect the success of pregnancy. There is significant evidence that exposure to BPA can lead to adverse reproductive outcomes such as intrauterine growth restriction (IUGR) [23] and preeclampsia (PE) [24], major pregnancy complications that can lead to morbidity and mortality in mother and fetus [25]. Because IUGR as well as PE are associated with reduced utero-placental blood flow [26], we hypothesized that BPA could affect uterine vascular function with consequent alterations of uterine hemodynamics and of pregnancy outcomes. Our results demonstrated, for the first time, that BPA interferes with UAs features, in particular, reduced vessel diameter, *NOS3* expression and compromised normal development of the fetoplacental unit. Taken together, our data suggest the adverse impact of BPA in pregnancy, underline UAs as specific targets for BPA and highlight the critical role that it may play in pregnancy success.

## 2. Results

### 2.1. BPA Impact on Fetal and Placental Outcomes

BPA was tested in the concentration range of 2.5–250 ug/kg/day, at lower and higher concentrations than the tolerable daily intake (TDI) of 50 ug/kg/day proposed by the U.S. Environmental Protection Agency [27].

Data on the effects of BPA exposure on the number of fetuses, fetal weight, and placental weight are summarized in Table 1. Treatment with BPA did not affect the number of fetuses, which was similar in control vs. BPA-treated rats. Fetal weights were significantly (*p* < 0.05) higher in rats treated with the lowest BPA concentration (2.5 µg/kg/day) vs. control. In contrast, placental weights were significantly lower (*p* < 0.01) in rats treated with either 2.5 or 250 µg/kg/day of BPA. Similarly, the ratio between placental and fetal weights was lower in rats treated with 2.5 (*p* < 0.001) or 250 µg/kg/day of BPA (*p* < 0.001).

### 2.2. BPA Impact on Maternal Uterine Arteries

**Figure 1:**
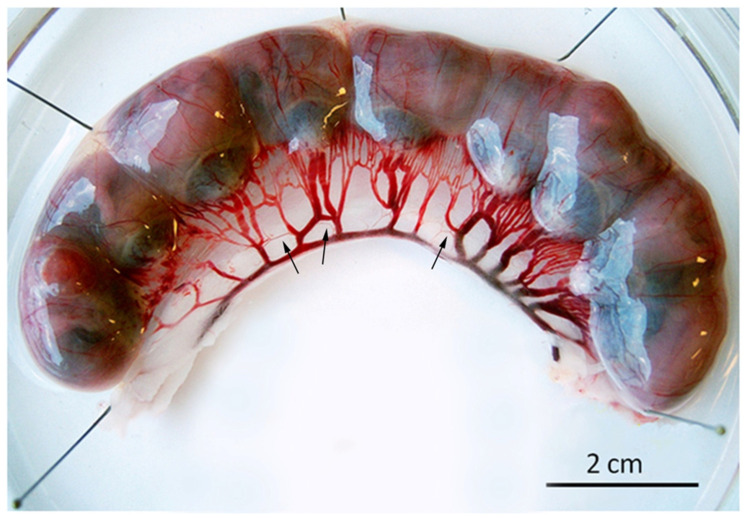
Image of one uterine horn from a pregnant rat showing several fetoplacental units and the maternal vasculature. Arrows indicate the arcuate UAs used in this study.

A second series of data concerns the influence of BPA on the function of maternal UAs in pregnant rats (see the image of uterine horn showing maternal UAs in Figure 1).

**Figure 2:**
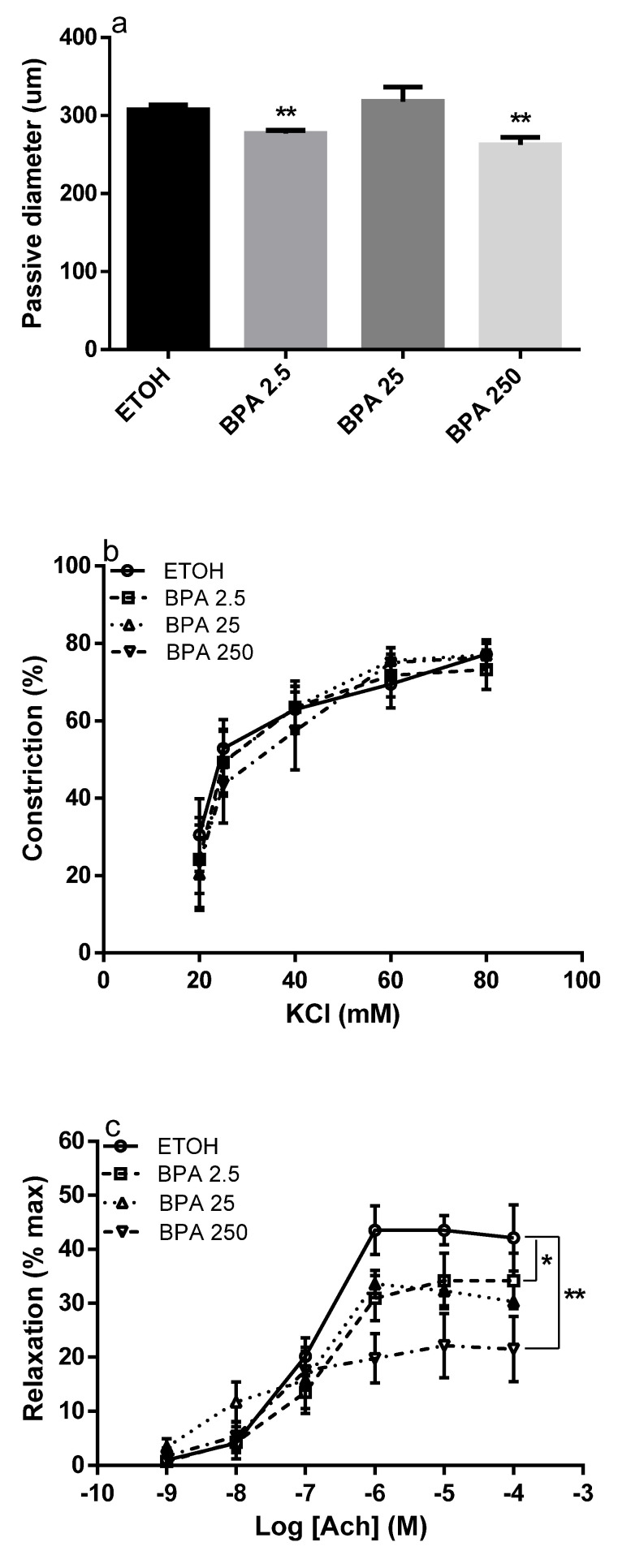

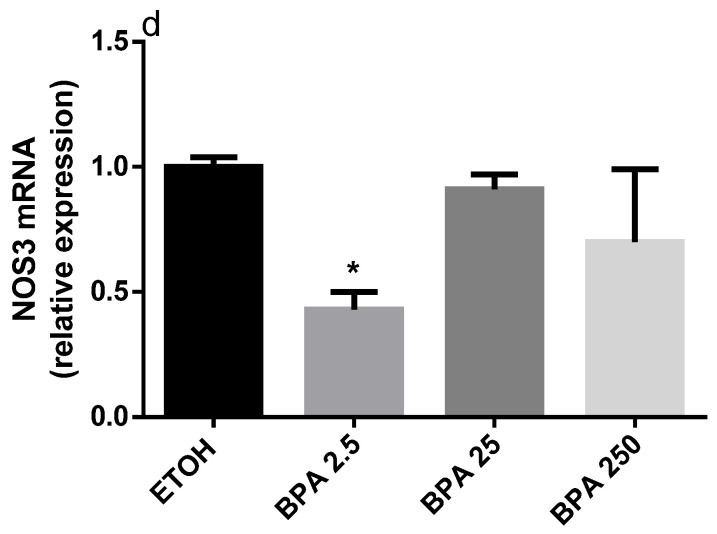
Effect of bisphenol A (BPA) on uterine arteries. The graphics show the passive diameter at 50 mmHg of intraluminal pressure (**a**); the KCl-induced contraction (**b**); the acetylcholine-relaxation (**c**); and the expression of *NOS3* (**d**) in UAs isolated from pregnant rats treated with BPA vehicle, ethanol (ETOH, *n* = 6) or with BPA at different concentrations expressed in µg/kg/day: 2.5 (*n* = 7), 25 (*n* = 8) and 250 (*n* = 6). The data are reported as Mean ± SEM, *n* = number of arteries, * *p* < 0.05, ** *p* < 0.01. Differences in responses between groups were determined by one-way ANOVA. The area under the curve was considered in Figure 2b,c.

In pregnant rats treated with BPA the UAs passive diameters (absence of vascular tone) were smaller than in control rats, with a significant decrease at 2.5 and 250 µg /kg/day (** *p* < 0.01), (Figure 2a).

To find out probable effects of BPA on UAs’ reactivity we individually tested the vasoagents KCl and acetylcholine on UAs isolated from both control and BPA rats. The constriction induced by KCl was similar in both groups of animals, (Figure 2b). Conversely, the relaxation caused by acetylcholine was reduced in UAs from rats treated with BPA compared to controls, with a significant effect at both 2.5 (*p* < 0.05) and 250 µg/kg/day (*p* < 0.01) BPA concentrations, (Figure 2c).

Because acetylcholine-relaxation is mediated mainly by nitric oxide (NO), to determine if the impairment induced by BPA involved the synthesis of NO, we assessed the *NOS3* expression. Interestingly, BPA treatment reduced the expression of *NOS3* in UAs with a large and significant decrease in mRNA levels at the lowest concentration tested, (Figure 2d).

**Figure 3:**
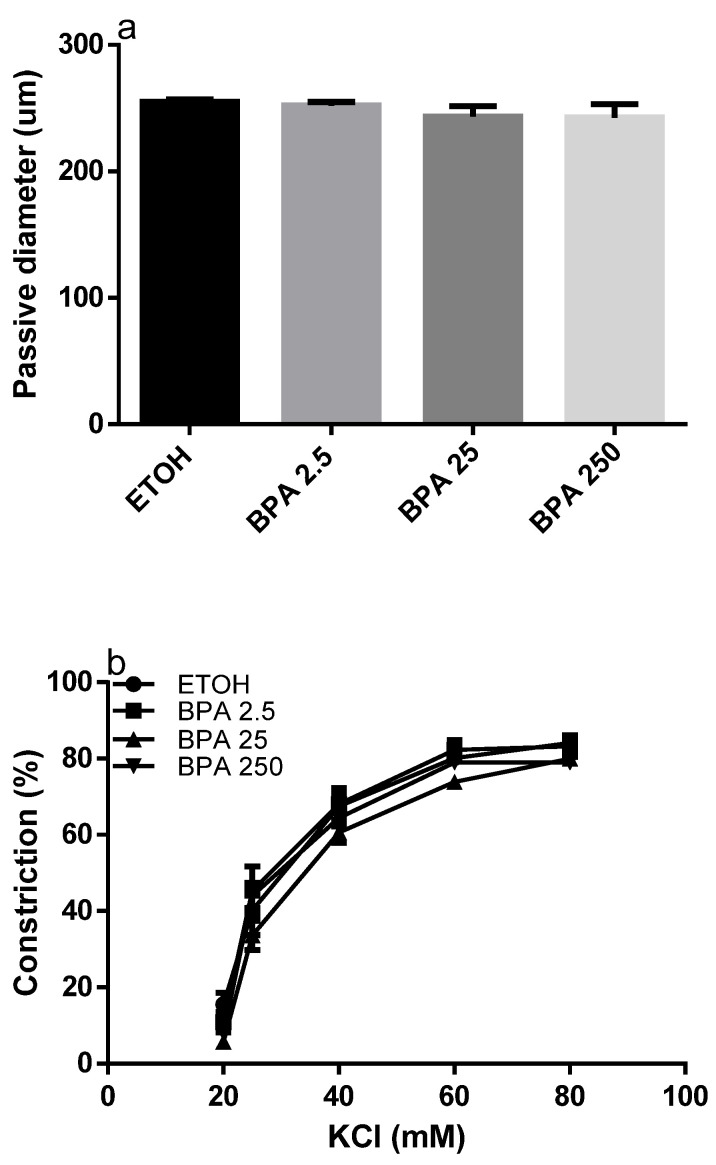

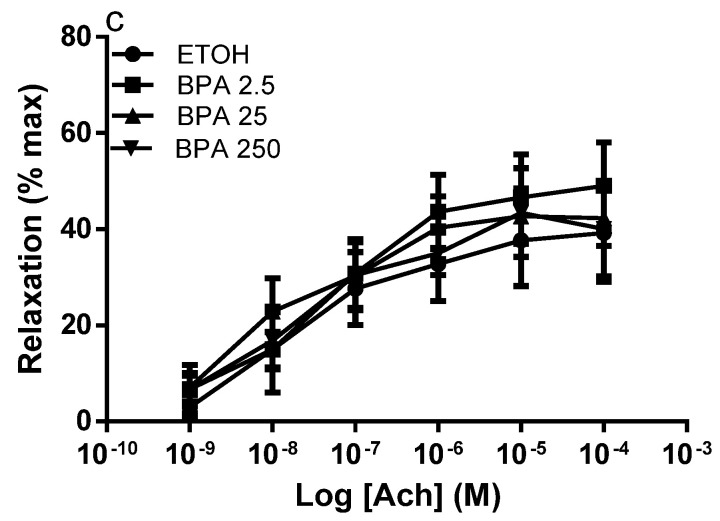
Effect of BPA on mesenteric arteries. The graphics show the passive diameter at 50 mmHg of intraluminal pressure (**a**), the KCl-induced contraction (**b**), and acetylcholine induced relaxation (**c**) in MAs isolated from pregnant rats treated with BPA vehicle (ETOH, *n* = 6), or with BPA at different concentrations expressed in µg/kg/day: 2.5 (*n* = 7), 25 (*n* = 8) and 250 (*n* = 6). Data are reported as Mean ± SEM and *n* = number of arteries. Data analysis was determined by one-way ANOVA. The area under the curve was considered in Figure 3b,c.

To determine if BPA affects the systemic circulation, we also considered its effects on MAs. As shown in Figure 3, passive diameter (a), KCl-constriction (b) and acetylcholine-relaxation (c) were unaffected in BPA-exposed animals with respect to controls.

**Figure 4:**
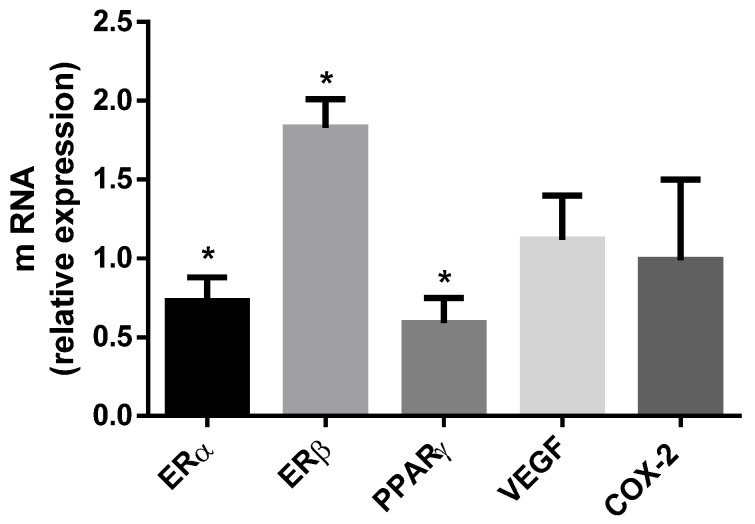
Effect of BPA on expression of selected genes. Graphic shows the expression of estrogen receptor alpha (*ERα*, *n* = 4); estrogen receptor beta (*ERβ*, *n* = 4); peroxisome proliferator activated receptor gamma (*PPARγ*, *n* = 4); vascular endothelial growth factor (*VEGF*, *n* = 5) and cyclooxygenase-2 (*COX-2*, *n* = 4) in UAs isolated from rats treated with BPA 2.5 µg /kg/day. The expression values are shown as fold of control group set as 1. Data are reported as Mean ± SEM and *n* = number of arteries, * *p* < 0.05. Data analysis was determined by one-way ANOVA.

To gain a first insight into the possible mechanisms of action of BPA on UAs, expression of selected genes that represent potential targets for BPA and are involved in endothelial functions were evaluated: *ERα* and estrogen receptors β (*ERβ*), *PPARγ*, the angiogenic cytokine Vascular endothelial growth factor (*VEGF*) and the inflammatory enzyme cyclooxygenase-2 (*COX-2*). The results, displayed in Figure 4, show that BPA induced significant downregulation of mRNA levels for *ERα* and *PPARγ* and upregulation of *ERβ*, whereas *VEGF* and *COX-2* were unaffected.

## 3. Discussion and Conclusions

Our results showed that BPA interferes with features of UAs of pregnant rats; in particular, it reduced lumen diameter and synthesis of NO, and compromised the normal fetoplacental development. In contrast, BPA did not affect the MAs (systemic vasculature), indicating that the UAs (reproductive vasculature) represent a specific target for BPA.

BPA acted in a concentration-dependent manner, showing significant effects only at the lowest and highest concentrations, suggesting an “inverted U-shaped dose response”, typical of many EDCs [28,29].

Our results showed no effect of BPA on the fetal number in contrast with a study on mice showing that BPA reduced the number of embryos [30]. The difference could be due to the BPA concentration used which was much higher (40 times more) than in our study.

Our data also showed that in animals treated with BPA, the ratio of placental/fetal weights, an expression of placental efficiency, was reduced, suggesting that BPA impairs the normal development of the fetoplacental unit, and compromises pregnancy outcomes. Similar results were reported in other studies in pregnant mice and in cultured placental cells where the treatment with BPA induced placental degenerative changes [30,31]. Placental dysfunction can consequently translate in vivo with different adverse pregnancy outcomes such as PE, IUGR or early termination of pregnancy [31].

In our study, although BPA reduced the weight of the placenta, the fetal weights increased. This effect may be explained by a compensatory action of the placenta, as reported in a previous publication demonstrating that BPA increased placental glucose transfer [19]. Several other reports have shown that a small placenta is a predictor of clinical complications such as maternal hypertension, gestation diabetes and fetal distress [32,33,34,35,36]. Although in the present study the possible association of a smaller placenta with maternal disease in BPA-exposed rats was not evaluated, this possibility requires further investigation.

Normal growth of the placenta depends on utero-placental blood flow that increases progressively and significantly [37] during pregnancy because of the circumferential growth of the uterine vasculature [38]. Therefore, we investigated the effect of BPA on the uterine vasculature. Our data showed that BPA reduced the passive diameter of UAs, which could partly explain the smaller placentas observed in rats treated with BPA.

Vascular tone is critically influenced by NO derived from NOS3 activity, that contributes to uterine quiescence and controls uteroplacental blood flow [39]. A reduced NO bioavailability is involved in the pathophysiological alterations of PE [40].

Our results show that in UAs BPA decreased acetylcholine-induced relaxation (known to be mediated by NO). Accordingly, a decrease in expression of *NOS3* was observed in rats treated with BPA. It suggests an inhibition of NO production by BPA, although measuring of phosphorylated and total *NOS3* ratio as well as of *NOS3* dimer would have been necessary to confirm this conclusion. In addition, BPA could have affected the smooth muscle cell (SMC) response to NO which could have been explored by testing SNP-induced relaxation. Inhibition of NO production or reducted SMC response to NO by BPA may explain the smaller UAs observed in BPA-treated animals.

Our results agree with previous studies on carotid arteries from mice and in cultured macrophage cells that showed an impairment of NO production by BPA [41]. In support of the crucial role that NO plays in the process of UAs dilation during pregnancy, several studies have shown a significant upregulation of *NOS3* expression and increase of NO in UAs from pregnant animals [42,43]. In addition, inhibition of NO is associated with smaller UA diameters [44,45]. The reduced production of NO, known also as endothelial dysfunction, is a major hallmark of PE, a major complication of pregnancy that is associated with maternal and fetal morbidity and mortality [46].

Although the mechanisms underlying pregnancy-associated uterine vasodilation are not fully understood, some regulator factors have been suggested to play an important role. In particular, estrogens, which have elevated levels in plasma during pregnancy, induce UA vasodilation through *NOS3* upregulation [47,48,49,50]. In addition, other factors such as *PPARγ* [51,52,53] and *VEGF* [54] can regulate the expression of *NOS3* in UA vasodilation associated with pregnancy. Interestingly, cross talk between *ERs*, *PPARγ* and *VEGF* has been reported [55,56].

In our experimental model BPA interferes with the expression of both *ERs* and *PPARγ*, while it did not affect *VEGF* expression. BPA as an endocrine disruptor can exert its action by a variety of signaling pathways, depending on the cell type, animal model and exposure conditions [57]. In UAs the reduction of *ERα* and of *PPARγ* could be the signal pathways by which BPA may interfere with UA features and cause pregnancy complications. Deregulation of *ERs* and *PPARγ* during pregnancy causes intrauterine growth restriction and PE [58,59].

We also considered the effect of BPA on *COX-2*, the isoform of the COX enzyme responsible for producing prostaglandins and reactive oxygen species that reduce the bioavailability of NO [60,61,62,63]. Our results showed that BPA did not affect the *COX-2* expression, suggesting it is not involved in the molecular mechanism underlying BPA’s effects.

The present study suggests that maternal exposure to BPA increases the risk for endothelial dysfunction in UAs through a variety of potential mechanisms and plays a pivotal role in the progression of pregnancy outcomes.

In contrast, BPA did not affect MAs, suggesting that it acts in a vascular-dependent manner that could be due to different vascular bed adaption to pregnancy [64,65].

In conclusion, our data showed for the first time that maternal exposure to BPA specifically targets uterine-placental blood flow, impairing fetoplacental development. Specific molecular mechanisms including a decrease in the expression of *NOS3*, *ERα* and *PPARγ* underlie the negative effects of BPA.

In addition to revealing a new mechanism of action of BPA, this study is further evidence of the harmful effects of this chemical for pregnancy and fetal development. Given the extensive knowledge on the distribution of BPA in the environment and the contamination of individuals with this chemical, it appears urgent to solicit the interest of the public, including non-professionals, to limit exposure to BPA in fertile or pregnant women, to ensure the health of future generations.

## 4. Material and Methods

### 4.1. Animals and Treatments

Experiments were performed on female Sprague Dawley rats (Envigo, Udine, Italy at 8 weeks of age. Rats were divided into four groups treated with BPA: (1) 2.5 μg/kg/day (*n* = 8); (2) 25 μg/kg/day (*n* = 8); (3); 250 μg/kg/day (*n* = 8) and (4) with vehicle (ethanol; *n* = 8). BPA or ethanol (0.04‰ *v*/*v*) were added to the drinking water for 30 days before pregnancy, and for the first 20 days of pregnancy. Pregnant rats were obtained by placing a female in estrus with a fertile male overnight; detection of spermatozoa using a vaginal smear on the following morning was used to confirm day 1 of pregnancy. Few animals, independently of the treatment, did not mate. All rats were housed individually in the animal care facility, maintained under controlled conditions on a 12-h light/dark photoperiod and provided with commercial chow and tap water ad libitum.

Animals used for experimental purposes on the day 20/22 of pregnancy were euthanized with isoflurane inhalation followed by decapitation with a small animal guillotine. All experiments were conducted in accordance with the European Guidelines for the care and use of laboratory animals (Directive 2010/63/EU) and were approved by the local ethical committee of the University of Calabria and the Italian Ministry of Health (n.74/2018-PR).

### 4.2. Reproductive Performance

Immediately after an animal was euthanized, the uterus and its vasculature were removed en bloc and positioned in a petri dish containing cold (4 °C) HEPES physiological saline solution (HEPES-PSS). Each fetus and placenta were carefully dissected away from the uterus and weighed without membranes and umbilical cords.

### 4.3. Isolated Vessel Preparation

Arcuate UAs and third-order MAs having similar diameters (250–300 µm at 50 mmHg), were isolated, respectively, from the mesometrium and mesentery. Arteries were dissected free of surrounding adipose and connective tissue and transferred to the chamber of a small vessel arteriograph (Instrumentation and Model Facility, University of Vermont, Burlington, VT, USA). Vessels were pressurized to 50 mmHg (a pressure that approximates in vivo conditions) using a pressure-servo system (Living Systems Instrumentation, Burlington, VT, USA), and continuously superfused with HEPES-PSS at 37 °C and pH = 7.4. Lumen diameter was continuously recorded by data-acquisition software (Ionoptix, Westwood, MA, USA).

### 4.4. Reactivity Study

Arteries were equilibrated for 30–45 min in HEPES-PSS and tested with increasing concentration of KCl (20–80 mM) then rinsed with HEPES-PSS. The same arteries were re-equilibrated and pre-constricted with KCl to produce a 40%–50% reduction in lumen diameter [66] prior to exposure to acetylcholine in a range of concentrations between 10^−9^ and 10^−5^ M. At the end of each experiment, vessels were treated with relaxing solution containing the L-type Ca^2+^ channel blocker diltiazem (10 µM), and the phosphodiesterase inhibitor, papaverine (100 µM) to assure maximal vasodilation, allowing us to record passive lumen diameter.

### 4.5. RNA Isolation and Gene Expression

After tissue homogenization, total RNA was isolated by the acid phenol–chloroform procedure using the Trizol reagent following the manufacturer’s instructions (Sigma-Aldrich Corp. Milan, Italy). RNA was dissolved in water and quantified, and the first strand cDNA synthesized in transcriptase buffer from 1 µg of total RNA using 200 U RevertAid H-Minus M-MuLV Reverse Transcriptase (ThermoFisher Scientific, Milano, Italy), 200 ng of oligo(dT)18-mer, 1 mM dNTPs (ThermoFisher Scientific, Milano, Italy), 100 U RNase inhibitor (ThermoFisher Scientific, Milano, Italy) in a final volume of 20 µL. Quantitative RT-PCR was performed in triplicates in a final volume of 15 µL containing 0.3 µM of each primer, 1× SybrGreen PCR Master Mix and samples were run and analyzed by CFX96 real-time system (Bio-Rad Laboratories, Monza, Italy) using the thermal protocol described previously [67]. Primer pairs and assay conditions are reported in Table S1. The relative quantity of target mRNA was calculated by using the comparative Cq method and normalized for the expression of glyceraldehyde 3-phosphate dehydrogenase (GAPDH). Data are reported as relative quantity of mRNA (relative expression) with respect to controls [68].

It is noteworthy that all primers included in this study were tested to determine the amplification efficiency range between 90%–110%. Briefly, control (ETOH) RNA was subjected to serial five-fold dilutions (not diluted; 1:5; 1:25; 1:125; 1:625; 1:3125) and quantitative RT-PCR was run in triplicates following the same protocol indicated above in this section.

### 4.6. Chemicals and Solutions

HEPES-PSS solution consists of the following components in mM: 141.8 NaCl; 4.7 KCl; 1.7 MgSO_4_; 0.5 EDTA; 2.8 CaCl_2_; 10.0 HEPES; 1.2 KH_2_PO_4_; 5.0 Glucose. Chemicals for HEPES PSS solutions were purchased from Fisher Scientific (Rodano, Italy). BPA, acetylcholine, papaverine, diltiazem, were purchased from Sigma Chemical Co. (Milano, Italy).

### 4.7. Statistical Analysis

Constriction to KCl was calculated as a reduction of the diameter of the vessels after equilibration and expressed as a percentage of initial diameter. Relaxation to acetylcholine was expressed as a percentage of the fully relaxed diameter (at 50 mmHg) determined in relaxing solution. We compared the area under the dose–response curve (AUC) for the different experimental conditions. All data are presented as mean ± SEM, where *n* is the number of arteries (also equal to the number of animals). Differences between groups were determined by one-way analysis of variance (ANOVA) (not repeated measures), with significance at *p* < 0.05.

## Figures and Tables

**Table 1:** Pregnancy outcomes.

	Control (*n* = 7)	BPA 2.5 µg/kg/day (*n* = 6)	BPA 25 µg/kg/die (*n* = 8)	BPA 250 µg/kg/die (*n* = 7)	*p* Value
**Fetal** **Number** (***n***)	13.0 ± 0.79	13.0 ± 0.89	13.1 ± 0.81	13.1 ± 0.94	NS
**Fetal** **Weight** (**g**)	2.15 ± 0.02	2.34 ± 0.11 *	2.13 ± 0.03	2.19 ± 0.02	BPA2.5 vs. Control * *p* = 0.0158
**Placental** **Weight** (**g**)	0.50 ± 0.01	0.48 ± 0.01 **	0.51 ± 0.01	0.45 ± 0.003 **	BPA2.5 vs. Control ** *p* = 0.0066 BPA250 vs. Control ** *p* = 0.0050
**Ratio Placental** **Fetal Weight**	0.23 ± 0.004	0.20 ± 0.006 ***	0.24 ± 0.008	0.21 ± 0.003 ***	BPA2.5 vs. Control *** *p* = 0.0008 BPA250 vs. Control *** *p*= 0.0002

* *p* < 0.05; ** *p* < 0.01; *** *p* < 0.001.

## Data Availability

Data available on request due to privacy.

## Supplementary Materials

The following are available online at https://www.mdpi.com/article/10.3390/ijms22136912/s1.
