# Supplementary material for: Bisphenol a Interferes with Uterine Artery Features and Impairs Rat Feto-Placental Growth
Source: Int J Mol Sci. 2021 Jun 27;22(13):6912. doi: 10.3390/ijms22136912 (PMC8268965; doi:10.3390/ijms22136912)
Supplement: Supplementary file 1 [file ijms-22-06912-s001.zip › ijms-1238728-supplementary.pdf]

Supplementary Material

Table S1. Primer pairs used for RT-Q-PCR analysis.

Primer Name	Primer Sequence (5′→3′)	Annealing Temperature (°C)	Product Lenght (bp)	Accession ID
GAPDH Fwd	GACCCCTTCATTGACCTCAAC	60	136	DQ403053
GAPDH Rev	CGCTCCTGGAAGATGGTGATGGG			
PPARγ Fwd	CGGAGTCCTCCCAGCTGTTCGCC	60	116	Y12882
PPARγ Rev	GGCTCATATCTGTCTCCGTCTTC			
ERα Fwd	CATCGATAAGAACCGGAGGA	60	150	AB477039
ERα Rev	TCTGACGCTTGTGCTTCAAC			
ERβ Fwd	GAAGCTGAACCACCCAATGT	60	150	AB190770
ERβ Rev	CAATCATGTGCACCAGTTCC			
VEGF Fwd	TTTCGGGAACTAGACCTCTCACC	60	109	NC_005108.4
VEGF Rev	CTTCATGTCAGGCTTTCTGGATT			
COX Fwd	TGTATGCTACCATCTGGCTTCGG	57	94	AF233596.1
COX Rev	GTTTGGAACAGTCGCTCGTCATC			

Primers for NOS3 were purchased from Bio-rad (UniqueAssayID: qRnoCID0005021).
